# Hydro­nium perchlorate–dibenzo-18-crown-6 (1/1): monoclinic polymorph

**DOI:** 10.1107/S1600536810048622

**Published:** 2010-11-27

**Authors:** Michaela Pojarová, Karla Fejfarová, Emanuel Makrlík

**Affiliations:** a Institute of Physics, AS CR, v.v.i., Na Slovance 2, 182 21 Praha 8, Czech Republic; b Faculty of Applied Sciences, University of West Bohemia, Husova 11, 306 14 Pilsen, Czech Republic

## Abstract

The asymmetric unit of the title compound, H_3_O^+^·ClO_4_^−^·C_20_H_24_O_6_, contains two mol­ecules/ions of each species. Both dibenzo-18-crown-6 mol­ecules have a complexed hydro­nium ion inside their cavity with O—H⋯O and O—H⋯(O,O) links between the two species. The associated perchlorate anions also accept O—H⋯O hydrogen bonds from the hydro­nium ion. Both crown ether mol­ecules are present in a butterfly conformation with approximate *C*_2v_ symmetry and their cavities are closed by the benzene ring of a neighbouring mol­ecule. The packing is consolidated by C—H⋯O and C—H⋯π inter­actions.

## Related literature

For the triclinic polymorph of the title compound, see: Chekhlov (2007 ▶).
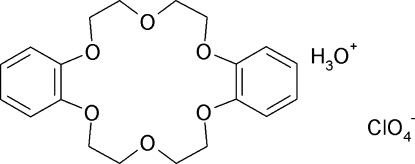


## Experimental

### 

#### Crystal data

H_3_O^+^·ClO_4_^−^·C_20_H_24_O_6_
*M* *_r_* = 478.9
Monoclinic, 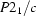

*a* = 8.6586 (1) Å
*b* = 26.7718 (3) Å
*c* = 19.1518 (2) Å
β = 100.0011 (10)°
*V* = 4372.05 (8) Å^3^
*Z* = 8
Cu *K*α radiation
μ = 2.09 mm^−1^
*T* = 124 K
0.26 × 0.18 × 0.13 mm

#### Data collection

Oxford Diffraction Xcalibur Atlas Gemini ultra diffractometer
Absorption correction: multi-scan (*CrysAlis RED*; Oxford Diffraction, 2008 ▶) *T*_min_ = 0.098, *T*_max_ = 1.000
36184 measured reflections
6865 independent reflections
5283 reflections with *I* > 3σ(*I*)
*R*_int_ = 0.048

#### Refinement

*R*[*F* ^2^ > 2σ(*F* ^2^)] = 0.051
*wR*(*F* ^2^) = 0.124
*S* = 2.07
6865 reflections
595 parameters
H atoms treated by a mixture of independent and constrained refinement
Δρ_max_ = 0.49 e Å^−3^
Δρ_min_ = −0.32 e Å^−3^

### 

Data collection: *CrysAlis CCD* (Oxford Diffraction, 2008 ▶); cell refinement: *CrysAlis RED* (Oxford Diffraction, 2008 ▶); data reduction: *CrysAlis RED*; program(s) used to solve structure: *SIR2002* (Burla *et al.*, 2003 ▶); program(s) used to refine structure: *JANA2006* (Petříček *et al.*, 2006 ▶); molecular graphics: *DIAMOND* (Brandenburg & Putz, 2005 ▶); software used to prepare material for publication: *JANA2006* and *publCIF* (Westrip, 2010 ▶).

## Supplementary Material

Crystal structure: contains datablocks global, I. DOI: 10.1107/S1600536810048622/hb5736sup1.cif

Structure factors: contains datablocks I. DOI: 10.1107/S1600536810048622/hb5736Isup2.hkl

Additional supplementary materials:  crystallographic information; 3D view; checkCIF report

## Figures and Tables

**Table 1:** Hydrogen-bond geometry (Å, °) *Cg*1, *Cg*2, *Cg*3 and *Cg*4 are the centroids of the C31–C36, C11–C16, C21–C26 and C1–C6 rings, respectively.

*D*—H⋯*A*	*D*—H	H⋯*A*	*D*⋯*A*	*D*—H⋯*A*
O21—H1⋯O3	1.14 (3)	1.64 (3)	2.763 (3)	168 (3)
O21—H1⋯O4	1.14 (3)	2.39 (3)	2.835 (3)	101 (2)
O21—H7⋯O17	1.22 (4)	1.73 (4)	2.945 (4)	171 (3)
O22—H8⋯O9	1.20 (3)	1.66 (3)	2.802 (3)	157 (3)
O22—H8⋯O10	1.20 (3)	2.29 (3)	2.837 (3)	104.4 (19)
O21—H9⋯O4	1.09 (3)	2.40 (3)	2.835 (3)	102 (2)
O21—H9⋯O5	1.09 (3)	1.87 (3)	2.910 (3)	159 (3)
O21—H9⋯O6	1.09 (3)	2.47 (4)	2.967 (3)	107 (2)
O22—H10⋯O11	1.05 (3)	1.90 (3)	2.840 (3)	149 (3)
O22—H10⋯O12	1.05 (3)	2.34 (3)	2.895 (3)	112 (2)
C5—H5⋯O18^i^	0.96	2.52	3.479 (3)	177
C8—H8*b*⋯O19	0.96	2.55	3.416 (3)	150
C15—H15⋯O13^ii^	0.96	2.42	3.369 (3)	169.02
C20—H20*b*⋯O16^iii^	0.96	2.43	3.165 (3)	134
C35—H35⋯O15^ii^	0.96	2.59	3.278 (4)	129
C38—H38*b*⋯O19^iv^	0.96	2.50	3.447 (3)	168
C17—H17*a*⋯*Cg*1	0.96	2.87	3.704 (3)	146
C37—H37*b*⋯*Cg*2^v^	0.96	2.99	3.825 (3)	146
C13—H13⋯*Cg*3	0.96	3.20	4.070 (3)	150
C33—H33⋯*Cg*4^v^	0.96	3.00	3.899 (3)	156

Symmetry codes: (i) 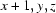; (ii) 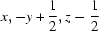; (iii) 
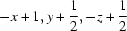
; (iv) 
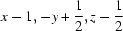
; (v) 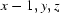.

**Fig. 1.**
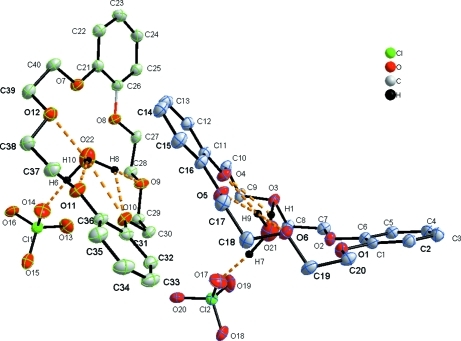
View of the asymmetric unit. The elipsoids are show with 50% probability and hydrogen atoms were omitted for better clarity.

**Fig. 2.**
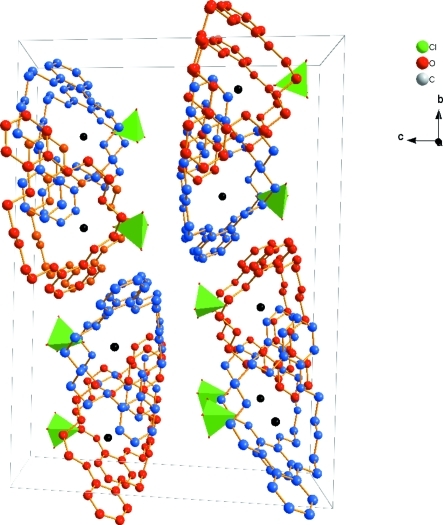
View along the a axis. The crown ether molecules form penentrating infinite channels filled with hydronium ions.

**Fig. 3.**
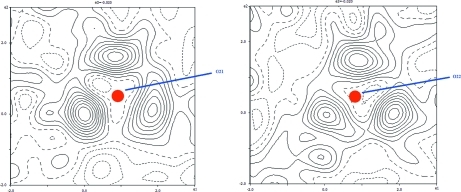
Difference electron density maps of hydronium groups.

## supplementary crystallographic information

### Comment

The crystal structure of dibenzo-18-crown-6 hydronium perchlorate was previously published by A.N.Chekhlov (2007). The published structure determined at room temperature is triclinic(space group P-1, a = 8.582 Å, b = 10.486 Å, c = 26.293 Å, α = 79.45°,β = 82.00° and γ = 79.36°, V =2272.5 Å) with asymmetric unit consisting of two independent molecules of macrocycle with complexed hydronium ions. The neutrality of the compound is ensured by two perchlorate anions. The data of crystal structure, presented in this paper, were collected at room temperature (testing stage) and at 120 K (final data collection). We found the complex monoclinic, *P*2_1_/*c* space group, with unit-cell parameters a = 8.6535 Å, b = 26.7823 Å, c = 19.1707 Å, β = 99.9987° and doubled unit cell volume V = 4372.05 Å^3^. The difference between both structures is in their system of hydrogen bonds. In Chekhlov's structure, the hydronium ion is held by three hydrogen bonds inside the crown cavity. In presented structure, hydronium ion and crown-ether form only two hydrogen bonds. The third hydrogen atom of hydronium ion is shared with perchlorate anion which makes it to point out of the cavity. This hydrogen bond causes that the perchlorate anions are not disordered as it was observed in Chekhlov's structure. Consequently, sharp maxima in difference Fourier map could be used for localizing hydrogen positions in both oxonia cations (Fig 3) and the found hydrogen positions could be refined without restraints. The distance between hydronium and oxygen atoms in macrocycles are 1.637 Å (O21—H1···O3) and 1.864 Å (O21—H9···O5) for one crownether molecule and 1.895 Å (O22—H10···O11) and 1.661 Å (O22—H8···O9) for the other one. The length of hydrogen bond between hydronium ion and perchlorate is 1.732 Å (O21—H7···O17) and 1.687 Å (O22—H6···O14). The distances between hydrogen atoms and oxygen atoms in hydronium correspond to the extent of their participation in hydrogen bonding system: O—H distance close (but still longer) to the standard value 0.983 Å has been only found for the weakest hydrogen bond O22—H10···O11. For stronger hydrogen bonds O—H distance becomes significantly longer, taking the maximum value 1.29 (4) for O22—H6···O14. The O—H and corresponding H···O distances for oxonia are summarized in Table 2. The hydronium ions are enclosed in the crown-ether cavities by phenyl ring of neighbouring molecules. This arrangement is stabilized due to CH-π interactions between phenyl rings and CH_2_ groups of crownether (the distance between centroid of phenyl ring C11→C16 and H37*b* in ethylen group is 2.989 Å and between centriod of phenyl ring C31→C36 and H17*a* in ethylen group is 2.870 Å) and due to the face-to-edge orientation of phenyl rings (distance between the centriod of phenyl ring C21→C26 and H13 of phenyl ring C11→C16 is 3.207 Å and between the centriod of phenyl ring C1→C6 and H33 in phenyl ring C31→C36 is 3.004 Å).

### Experimental

Dibenzo-18-crown-6, perchloric acid and acetonitrile were purchased by Fluka. Crystals were prepared by slow evaporation of equimolar mixture of dibenzo-18-crown-6 (0.05*M*) and perchloric acid (0.05*M*) in acetonitrile to yield colourless prisms of the title compound.

### Crystal data

**Table:**

H_3_O^+^·ClO_4_^−^·C_20_H_24_O_6_	*F*(000) = 2016
*M**_r_* = 478.9	*D*_x_ = 1.455 Mg m^−^^3^
Monoclinic, *P*2_1_/*c*	Cu *K*α radiation, λ = 1.5418 Å
Hall symbol: -P 2ybc	Cell parameters from 20984 reflections
*a* = 8.6586 (1) Å	θ = 3.3–62.5°
*b* = 26.7718 (3) Å	µ = 2.09 mm^−^^1^
*c* = 19.1518 (2) Å	*T* = 124 K
β = 100.0011 (10)°	Prism, colourless
*V* = 4372.05 (8) Å^3^	0.26 × 0.18 × 0.13 mm
*Z* = 8	

### Data collection

**Table:**

Oxford Diffraction Xcalibur Atlas Gemini ultra diffractometer	6865 independent reflections
Radiation source: Enhance Ultra (Cu) X-ray Source	5283 reflections with *I* > 3σ(*I*)
mirror	*R*_int_ = 0.048
Detector resolution: 10.3784 pixels mm^-1^	θ_max_ = 62.6°, θ_min_ = 3.3°
rotation method data acquisition using ω scans	*h* = −9→9
Absorption correction: multi-scan (*CrysAlis RED*; Oxford Diffraction, 2008)	*k* = −30→28
*T*_min_ = 0.098, *T*_max_ = 1.000	*l* = −22→21
36184 measured reflections	

### Refinement

**Table:**

Refinement on *F*^2^	198 constraints
*R*[*F*^2^ > 2σ(*F*^2^)] = 0.051	H atoms treated by a mixture of independent and constrained refinement
*wR*(*F*^2^) = 0.124	Weighting scheme based on measured s.u.'s *w* = 1/[σ^2^(*I*) + 0.0016*I*^2^]
*S* = 2.07	(Δ/σ)_max_ = 0.022
6865 reflections	Δρ_max_ = 0.49 e Å^−^^3^
595 parameters	Δρ_min_ = −0.32 e Å^−^^3^
0 restraints	

### Special details

**Table:**

Refinement. The refinement was carried out against all reflections. The conventional *R*-factor is always based on *F*. The goodness of fit as well as the weighted *R*-factor are based on *F* and *F*^2^ for refinement carried out on *F* and *F*^2^, respectively. The threshold expression is used only for calculating *R*-factors *etc*. and it is not relevant to the choice of reflections for refinement.All the H atoms were discernible in difference Fourier maps and could be refined to reasonable geometry. Despite of it the H atoms bonded to carbon atoms were constrained to ideal positions. The O—H distances and angles in hydronium ions were not restrained. The isotropic temperature parameters of hydrogen atoms were calculated as 1.2**U*_eq_ of the parent atom.The program used for refinement, Jana2006, uses the weighting scheme based on the experimental expectations, see _refine_ls_weighting_details.

### Fractional atomic coordinates and isotropic or equivalent isotropic displacement parameters (Å^2^)

**Table:**

	*x*	*y*	*z*	*U*_iso_*/*U*_eq_	
Cl1	0.25806 (7)	0.16885 (2)	0.35587 (3)	0.0248 (2)	
Cl2	0.74151 (7)	0.41184 (2)	0.38383 (4)	0.0261 (2)	
O1	1.0665 (2)	0.50098 (6)	0.15146 (9)	0.0252 (6)	
O2	1.2316 (2)	0.46140 (6)	0.26441 (9)	0.0238 (6)	
O3	1.1821 (2)	0.35876 (6)	0.28737 (9)	0.0270 (6)	
O4	0.9437 (2)	0.30634 (6)	0.19288 (9)	0.0249 (6)	
O5	0.7465 (2)	0.35281 (6)	0.09651 (9)	0.0271 (6)	
O6	0.8039 (2)	0.45403 (6)	0.06355 (9)	0.0243 (6)	
O7	0.5802 (2)	0.08959 (6)	0.11052 (9)	0.0263 (6)	
O8	0.7525 (2)	0.12376 (6)	0.22540 (9)	0.0243 (6)	
O9	0.7056 (2)	0.22401 (6)	0.27262 (9)	0.0268 (6)	
O10	0.4558 (2)	0.28100 (6)	0.19535 (10)	0.0267 (6)	
O11	0.2643 (2)	0.24193 (6)	0.08853 (9)	0.0284 (6)	
O12	0.3196 (2)	0.14350 (6)	0.03615 (10)	0.0266 (6)	
O13	0.4098 (2)	0.17956 (8)	0.39605 (12)	0.0404 (7)	
O14	0.2617 (2)	0.17463 (8)	0.28118 (10)	0.0400 (8)	
O15	0.1434 (2)	0.20252 (7)	0.37496 (10)	0.0327 (7)	
O16	0.2159 (2)	0.11841 (7)	0.36877 (11)	0.0348 (7)	
O17	0.7348 (2)	0.40931 (8)	0.30779 (10)	0.0399 (8)	
O18	0.6961 (2)	0.46121 (6)	0.40192 (10)	0.0298 (6)	
O19	0.8976 (2)	0.40152 (8)	0.41890 (12)	0.0452 (8)	
O20	0.6341 (2)	0.37595 (7)	0.40378 (11)	0.0345 (7)	
O21	0.9470 (3)	0.41200 (9)	0.20320 (14)	0.0495 (9)	
O22	0.4740 (3)	0.17637 (9)	0.17481 (13)	0.0478 (9)	
C1	1.2210 (3)	0.51595 (9)	0.16739 (14)	0.0213 (8)	
C2	1.2909 (3)	0.54843 (9)	0.12647 (14)	0.0249 (9)	
C3	1.4465 (3)	0.56198 (10)	0.14712 (14)	0.0273 (9)	
C4	1.5327 (3)	0.54337 (10)	0.20921 (15)	0.0272 (9)	
C5	1.4644 (3)	0.50926 (9)	0.25017 (14)	0.0251 (9)	
C6	1.3092 (3)	0.49536 (9)	0.22946 (14)	0.0218 (8)	
C7	1.3267 (3)	0.43350 (10)	0.32048 (13)	0.0243 (9)	
C8	1.2279 (3)	0.39347 (10)	0.34457 (14)	0.0271 (9)	
C9	1.0956 (3)	0.31719 (9)	0.30858 (14)	0.0277 (9)	
C10	1.0523 (3)	0.28207 (10)	0.24743 (13)	0.0251 (9)	
C11	0.8679 (3)	0.27715 (9)	0.13818 (14)	0.0235 (9)	
C12	0.8904 (3)	0.22609 (10)	0.13211 (14)	0.0263 (9)	
C13	0.8027 (3)	0.19963 (10)	0.07643 (15)	0.0295 (10)	
C14	0.6942 (3)	0.22368 (10)	0.02681 (16)	0.0315 (10)	
C15	0.6734 (3)	0.27535 (10)	0.03163 (15)	0.0300 (9)	
C16	0.7594 (3)	0.30152 (9)	0.08723 (14)	0.0249 (9)	
C17	0.6510 (3)	0.37942 (10)	0.03977 (14)	0.0264 (9)	
C18	0.6510 (3)	0.43353 (10)	0.05990 (14)	0.0267 (9)	
C19	0.8080 (3)	0.50524 (9)	0.08415 (14)	0.0264 (9)	
C20	0.9729 (3)	0.52437 (10)	0.09165 (14)	0.0251 (9)	
C21	0.7327 (3)	0.07209 (9)	0.12468 (14)	0.0224 (8)	
C22	0.7959 (3)	0.03910 (10)	0.08188 (14)	0.0268 (9)	
C23	0.9502 (3)	0.02291 (10)	0.10120 (15)	0.0295 (9)	
C24	1.0392 (3)	0.04011 (10)	0.16306 (15)	0.0285 (9)	
C25	0.9785 (3)	0.07432 (9)	0.20588 (15)	0.0262 (9)	
C26	0.8251 (3)	0.09029 (9)	0.18699 (14)	0.0232 (8)	
C27	0.8501 (3)	0.14721 (9)	0.28477 (13)	0.0237 (8)	
C28	0.7525 (3)	0.18269 (10)	0.31932 (14)	0.0272 (9)	
C29	0.6189 (3)	0.26055 (9)	0.30496 (15)	0.0277 (9)	
C30	0.5650 (3)	0.30146 (10)	0.25339 (14)	0.0258 (9)	
C31	0.3730 (3)	0.31430 (10)	0.14860 (14)	0.0247 (9)	
C32	0.3859 (3)	0.36568 (10)	0.15492 (15)	0.0291 (10)	
C33	0.2916 (3)	0.39643 (11)	0.10659 (15)	0.0336 (10)	
C34	0.1876 (4)	0.37606 (11)	0.05210 (16)	0.0362 (10)	
C35	0.1769 (4)	0.32457 (10)	0.04384 (16)	0.0329 (10)	
C36	0.2687 (3)	0.29363 (9)	0.09150 (14)	0.0247 (9)	
C37	0.1739 (3)	0.22021 (10)	0.02603 (14)	0.0287 (9)	
C39	0.3192 (3)	0.09096 (9)	0.04725 (14)	0.0269 (9)	
C38	0.1685 (3)	0.16499 (10)	0.03607 (15)	0.0291 (9)	
C40	0.4813 (3)	0.07089 (10)	0.04808 (14)	0.0255 (9)	
H2	1.231405	0.561714	0.083496	0.0299*	
H3	1.494768	0.584392	0.118191	0.0328*	
H4	1.63932	0.55383	0.224244	0.0326*	
H5	1.525077	0.49554	0.292542	0.0302*	
H12	0.966406	0.209052	0.166353	0.0316*	
H13	0.81811	0.164292	0.072615	0.0354*	
H14	0.632921	0.205091	−0.011045	0.0378*	
H15	0.599728	0.292466	−0.003542	0.036*	
H22	0.733316	0.027309	0.038698	0.0321*	
H23	0.994135	−0.000095	0.071569	0.0354*	
H24	1.144758	0.028339	0.17691	0.0342*	
H25	1.042704	0.086749	0.248246	0.0314*	
H32	0.459801	0.380137	0.192675	0.0349*	
H33	0.299694	0.432064	0.111515	0.0403*	
H34	0.121755	0.397456	0.019413	0.0435*	
H35	0.105429	0.310504	0.004858	0.0395*	
H7a	1.364134	0.455426	0.359401	0.0292*	
H7b	1.413162	0.41857	0.302888	0.0292*	
H8a	1.287265	0.376258	0.384371	0.0325*	
H8b	1.135985	0.408146	0.357785	0.0325*	
H9a	1.002067	0.329164	0.323545	0.0332*	
H9b	1.159082	0.299958	0.347238	0.0332*	
H10a	1.144849	0.272934	0.229166	0.0301*	
H10b	1.004393	0.252726	0.263034	0.0301*	
H17a	0.545754	0.366738	0.033141	0.0317*	
H17b	0.694493	0.375793	−0.002769	0.0317*	
H18a	0.578391	0.451419	0.02519	0.0321*	
H18b	0.6189	0.436793	0.105205	0.0321*	
H19a	0.772303	0.508381	0.128674	0.0317*	
H19b	0.741144	0.524343	0.04869	0.0317*	
H20a	1.01435	0.516511	0.049624	0.0302*	
H20b	0.973457	0.559875	0.098682	0.0302*	
H27a	0.932884	0.165285	0.268531	0.0285*	
H27b	0.893741	0.122157	0.318336	0.0285*	
H28a	0.661042	0.165685	0.329117	0.0326*	
H28b	0.812898	0.194786	0.362846	0.0326*	
H29a	0.684642	0.274275	0.345999	0.0333*	
H29b	0.529575	0.244913	0.319251	0.0333*	
H30a	0.514058	0.326949	0.27639	0.031*	
H30b	0.653423	0.315137	0.235919	0.031*	
H37a	0.222792	0.22752	−0.014119	0.0344*	
H37b	0.069301	0.233425	0.018916	0.0344*	
H39a	0.247671	0.07545	0.009571	0.0323*	
H39b	0.287414	0.084002	0.091848	0.0323*	
H38a	0.13522	0.157791	0.080279	0.0349*	
H38b	0.094171	0.150572	−0.001568	0.0349*	
H40a	0.478954	0.035064	0.049777	0.0306*	
H40b	0.519586	0.082359	0.006772	0.0306*	
H1	1.043 (4)	0.3869 (13)	0.2320 (19)	0.0594*	
H6	0.384 (4)	0.1733 (12)	0.2216 (18)	0.0574*	
H7	0.855 (4)	0.4148 (12)	0.2446 (19)	0.0594*	
H8	0.572 (4)	0.2039 (12)	0.2057 (18)	0.0574*	
H9	0.893 (4)	0.3913 (13)	0.156 (2)	0.0594*	
H10	0.421 (4)	0.1965 (13)	0.1302 (19)	0.0574*	

### Atomic displacement parameters (Å^2^)

**Table:**

	*U*^11^	*U*^22^	*U*^33^	*U*^12^	*U*^13^	*U*^23^
Cl1	0.0258 (3)	0.0217 (3)	0.0262 (3)	−0.0007 (3)	0.0022 (3)	0.0018 (3)
Cl2	0.0255 (3)	0.0245 (3)	0.0287 (4)	0.0012 (3)	0.0055 (3)	−0.0003 (3)
O1	0.0219 (10)	0.0248 (10)	0.0276 (10)	−0.0008 (8)	0.0004 (8)	0.0063 (8)
O2	0.0254 (10)	0.0211 (9)	0.0240 (10)	−0.0010 (8)	0.0020 (8)	0.0068 (8)
O3	0.0385 (11)	0.0212 (10)	0.0217 (10)	−0.0086 (8)	0.0057 (8)	−0.0004 (8)
O4	0.0308 (11)	0.0193 (9)	0.0222 (10)	0.0028 (8)	−0.0023 (8)	0.0007 (8)
O5	0.0345 (11)	0.0176 (9)	0.0258 (10)	0.0015 (8)	−0.0042 (8)	0.0004 (8)
O6	0.0259 (10)	0.0161 (9)	0.0295 (10)	0.0001 (8)	0.0010 (8)	−0.0017 (8)
O7	0.0255 (10)	0.0250 (10)	0.0268 (10)	0.0005 (8)	−0.0003 (8)	−0.0064 (8)
O8	0.0269 (10)	0.0213 (9)	0.0236 (10)	0.0012 (8)	0.0014 (8)	−0.0051 (8)
O9	0.0396 (11)	0.0189 (9)	0.0227 (10)	0.0054 (8)	0.0074 (8)	0.0015 (8)
O10	0.0341 (11)	0.0181 (9)	0.0257 (10)	−0.0008 (8)	−0.0009 (8)	−0.0012 (8)
O11	0.0358 (11)	0.0207 (10)	0.0259 (10)	0.0012 (8)	−0.0022 (9)	−0.0016 (8)
O12	0.0273 (10)	0.0207 (10)	0.0309 (11)	0.0011 (8)	0.0027 (8)	−0.0005 (8)
O13	0.0249 (11)	0.0429 (13)	0.0482 (14)	−0.0092 (9)	−0.0080 (10)	−0.0016 (10)
O14	0.0500 (14)	0.0495 (13)	0.0231 (11)	0.0046 (10)	0.0135 (10)	0.0041 (9)
O15	0.0371 (12)	0.0303 (11)	0.0304 (11)	0.0104 (9)	0.0048 (9)	−0.0022 (9)
O16	0.0360 (12)	0.0173 (10)	0.0503 (13)	−0.0042 (8)	0.0053 (10)	0.0068 (9)
O17	0.0541 (14)	0.0455 (13)	0.0241 (11)	−0.0078 (10)	0.0185 (10)	−0.0074 (9)
O18	0.0388 (12)	0.0193 (10)	0.0300 (11)	0.0057 (8)	0.0027 (9)	−0.0035 (8)
O19	0.0232 (11)	0.0461 (13)	0.0626 (16)	0.0111 (10)	−0.0032 (10)	0.0050 (11)
O20	0.0379 (12)	0.0254 (10)	0.0423 (12)	−0.0069 (9)	0.0127 (10)	0.0065 (9)
O21	0.0451 (14)	0.0437 (14)	0.0543 (16)	−0.0028 (11)	−0.0068 (12)	0.0096 (12)
O22	0.0459 (14)	0.0455 (14)	0.0467 (15)	0.0057 (11)	−0.0070 (12)	−0.0087 (11)
C1	0.0202 (13)	0.0173 (13)	0.0258 (14)	0.0009 (11)	0.0026 (11)	−0.0028 (11)
C2	0.0291 (15)	0.0199 (14)	0.0259 (15)	0.0013 (12)	0.0056 (12)	0.0015 (11)
C3	0.0301 (16)	0.0248 (15)	0.0276 (15)	−0.0035 (12)	0.0066 (12)	0.0018 (12)
C4	0.0256 (15)	0.0225 (14)	0.0334 (16)	−0.0006 (12)	0.0052 (12)	0.0005 (12)
C5	0.0280 (15)	0.0214 (14)	0.0255 (15)	0.0006 (12)	0.0031 (12)	−0.0002 (11)
C6	0.0247 (14)	0.0153 (13)	0.0262 (14)	−0.0015 (11)	0.0064 (11)	−0.0005 (11)
C7	0.0290 (15)	0.0231 (14)	0.0192 (14)	0.0018 (12)	−0.0005 (11)	0.0014 (11)
C8	0.0343 (16)	0.0257 (14)	0.0207 (15)	−0.0046 (12)	0.0030 (12)	−0.0009 (12)
C9	0.0345 (16)	0.0233 (14)	0.0244 (15)	−0.0069 (12)	0.0029 (12)	0.0047 (12)
C10	0.0299 (15)	0.0184 (13)	0.0255 (15)	−0.0004 (11)	0.0010 (12)	0.0065 (11)
C11	0.0291 (15)	0.0203 (14)	0.0213 (14)	−0.0042 (12)	0.0052 (12)	−0.0005 (11)
C12	0.0325 (16)	0.0212 (14)	0.0259 (15)	−0.0002 (12)	0.0066 (12)	0.0030 (12)
C13	0.0392 (17)	0.0189 (14)	0.0313 (16)	0.0001 (12)	0.0090 (13)	0.0005 (12)
C14	0.0364 (17)	0.0267 (16)	0.0297 (16)	−0.0060 (13)	0.0016 (13)	−0.0059 (13)
C15	0.0343 (17)	0.0247 (15)	0.0284 (16)	−0.0012 (13)	−0.0018 (13)	0.0011 (12)
C16	0.0299 (15)	0.0177 (14)	0.0264 (15)	−0.0018 (11)	0.0030 (12)	0.0013 (11)
C17	0.0280 (15)	0.0235 (14)	0.0252 (15)	0.0006 (12)	−0.0023 (12)	0.0052 (12)
C18	0.0226 (14)	0.0254 (15)	0.0296 (16)	−0.0002 (12)	−0.0026 (12)	0.0029 (12)
C19	0.0281 (15)	0.0183 (14)	0.0305 (16)	0.0036 (11)	−0.0014 (12)	−0.0019 (12)
C20	0.0309 (15)	0.0191 (14)	0.0236 (14)	0.0045 (12)	0.0000 (12)	0.0029 (11)
C21	0.0215 (14)	0.0190 (13)	0.0267 (15)	0.0018 (11)	0.0042 (11)	0.0031 (11)
C22	0.0329 (16)	0.0234 (15)	0.0235 (15)	−0.0008 (12)	0.0038 (12)	−0.0005 (12)
C23	0.0333 (16)	0.0263 (15)	0.0299 (16)	0.0043 (13)	0.0084 (13)	0.0002 (12)
C24	0.0262 (15)	0.0244 (15)	0.0351 (17)	0.0018 (12)	0.0060 (13)	0.0004 (12)
C25	0.0303 (15)	0.0222 (14)	0.0255 (15)	−0.0013 (12)	0.0030 (12)	0.0010 (12)
C26	0.0268 (15)	0.0179 (13)	0.0250 (15)	−0.0008 (11)	0.0046 (12)	−0.0015 (11)
C27	0.0261 (15)	0.0216 (14)	0.0213 (14)	−0.0035 (11)	−0.0020 (11)	0.0003 (11)
C28	0.0348 (17)	0.0242 (14)	0.0215 (14)	0.0006 (12)	0.0021 (12)	0.0011 (12)
C29	0.0341 (16)	0.0234 (14)	0.0263 (15)	0.0016 (12)	0.0070 (12)	−0.0051 (12)
C30	0.0298 (15)	0.0207 (14)	0.0262 (15)	−0.0025 (12)	0.0027 (12)	−0.0057 (11)
C31	0.0306 (16)	0.0230 (14)	0.0223 (14)	0.0015 (12)	0.0091 (12)	0.0019 (11)
C32	0.0374 (17)	0.0227 (15)	0.0296 (16)	−0.0008 (12)	0.0125 (13)	−0.0023 (12)
C33	0.0499 (19)	0.0211 (15)	0.0322 (17)	0.0052 (14)	0.0139 (15)	0.0014 (13)
C34	0.0502 (19)	0.0265 (16)	0.0329 (17)	0.0116 (14)	0.0096 (15)	0.0059 (13)
C35	0.0414 (18)	0.0289 (16)	0.0277 (16)	0.0040 (13)	0.0039 (14)	0.0001 (13)
C36	0.0320 (15)	0.0178 (14)	0.0248 (14)	0.0015 (12)	0.0067 (12)	−0.0001 (11)
C37	0.0306 (16)	0.0287 (15)	0.0240 (15)	0.0015 (12)	−0.0027 (12)	−0.0041 (12)
C39	0.0288 (15)	0.0221 (14)	0.0284 (15)	−0.0032 (12)	0.0010 (12)	−0.0019 (12)
C38	0.0244 (15)	0.0307 (15)	0.0300 (16)	0.0023 (12)	−0.0014 (12)	−0.0050 (13)
C40	0.0315 (15)	0.0212 (14)	0.0221 (14)	−0.0003 (12)	−0.0001 (12)	−0.0044 (11)

### Geometric parameters (Å, °)

**Table:**

Cl1—O13	1.4313 (19)	C13—C14	1.375 (4)
Cl1—O14	1.445 (2)	C13—H13	0.96
Cl1—O15	1.435 (2)	C14—C15	1.400 (4)
Cl1—O16	1.4315 (19)	C14—H14	0.96
Cl2—O17	1.449 (2)	C15—C16	1.380 (4)
Cl2—O18	1.4385 (19)	C15—H15	0.96
Cl2—O19	1.428 (2)	C17—C18	1.499 (4)
Cl2—O20	1.434 (2)	C17—H17a	0.96
O1—C1	1.379 (3)	C17—H17b	0.96
O1—C20	1.428 (3)	C18—H18a	0.96
O2—C6	1.372 (3)	C18—H18b	0.96
O2—C7	1.443 (3)	C19—C20	1.499 (4)
O3—C8	1.439 (3)	C19—H19a	0.96
O3—C9	1.438 (3)	C19—H19b	0.96
O4—C10	1.434 (3)	C20—H20a	0.96
O4—C11	1.379 (3)	C20—H20b	0.96
O5—C16	1.392 (3)	C21—C22	1.381 (4)
O5—C17	1.435 (3)	C21—C26	1.403 (3)
O6—C18	1.423 (3)	C22—C23	1.392 (4)
O6—C19	1.426 (3)	C22—H22	0.96
O7—C21	1.383 (3)	C23—C24	1.376 (4)
O7—C40	1.435 (3)	C23—H23	0.96
O8—C26	1.378 (3)	C24—C25	1.392 (4)
O8—C27	1.438 (3)	C24—H24	0.96
O9—C28	1.436 (3)	C25—C26	1.382 (4)
O9—C29	1.437 (3)	C25—H25	0.96
O10—C30	1.437 (3)	C27—C28	1.499 (4)
O10—C31	1.374 (3)	C27—H27a	0.96
O11—C36	1.385 (3)	C27—H27b	0.96
O11—C37	1.435 (3)	C28—H28a	0.96
O12—C39	1.423 (3)	C28—H28b	0.96
O12—C38	1.429 (3)	C29—C30	1.494 (4)
C1—C2	1.379 (4)	C29—H29a	0.96
C1—C6	1.409 (3)	C29—H29b	0.96
C2—C3	1.386 (4)	C30—H30a	0.96
C2—H2	0.96	C30—H30b	0.96
C3—C4	1.382 (4)	C31—C32	1.384 (4)
C3—H3	0.96	C31—C36	1.405 (4)
C4—C5	1.400 (4)	C32—C33	1.392 (4)
C4—H4	0.96	C32—H32	0.96
C5—C6	1.384 (4)	C33—C34	1.368 (4)
C5—H5	0.96	C33—H33	0.96
C7—C8	1.493 (4)	C34—C35	1.389 (4)
C7—H7a	0.96	C34—H34	0.96
C7—H7b	0.96	C35—C36	1.378 (4)
C8—H8a	0.96	C35—H35	0.96
C8—H8b	0.96	C37—C38	1.493 (4)
C9—C10	1.498 (4)	C37—H37a	0.96
C9—H9a	0.96	C37—H37b	0.96
C9—H9b	0.96	C39—C40	1.500 (4)
C10—H10a	0.96	C39—H39a	0.96
C10—H10b	0.96	C39—H39b	0.96
C11—C12	1.388 (4)	C38—H38a	0.96
C11—C16	1.393 (3)	C38—H38b	0.96
C12—C13	1.391 (4)	C40—H40a	0.96
C12—H12	0.96	C40—H40b	0.96
			
O13—Cl1—O14	109.58 (13)	O6—C19—C20	109.4 (2)
O13—Cl1—O15	110.37 (12)	O6—C19—H19a	109.4711
O13—Cl1—O16	109.51 (12)	O6—C19—H19b	109.4709
O14—Cl1—O15	108.66 (12)	C20—C19—H19a	109.4713
O14—Cl1—O16	108.79 (13)	C20—C19—H19b	109.4711
O15—Cl1—O16	109.90 (12)	H19a—C19—H19b	109.4972
O17—Cl2—O18	108.66 (12)	O1—C20—C19	109.0 (2)
O17—Cl2—O19	109.47 (14)	O1—C20—H20a	109.4717
O17—Cl2—O20	108.77 (12)	O1—C20—H20b	109.4714
O18—Cl2—O19	109.90 (12)	C19—C20—H20a	109.4707
O18—Cl2—O20	109.54 (12)	C19—C20—H20b	109.4714
O19—Cl2—O20	110.47 (12)	H20a—C20—H20b	109.9286
C1—O1—C20	116.4 (2)	O7—C21—C22	124.3 (2)
C6—O2—C7	116.22 (19)	O7—C21—C26	115.7 (2)
C8—O3—C9	111.9 (2)	C22—C21—C26	120.0 (2)
C10—O4—C11	117.51 (19)	C21—C22—C23	120.1 (2)
C16—O5—C17	116.21 (19)	C21—C22—H22	119.9264
C18—O6—C19	111.29 (19)	C23—C22—H22	119.9263
C21—O7—C40	117.2 (2)	C22—C23—C24	119.5 (3)
C26—O8—C27	116.48 (19)	C22—C23—H23	120.231
C28—O9—C29	111.6 (2)	C24—C23—H23	120.2312
C30—O10—C31	117.10 (19)	C23—C24—C25	121.0 (3)
C36—O11—C37	116.52 (19)	C23—C24—H24	119.4897
C39—O12—C38	111.8 (2)	C25—C24—H24	119.4882
O1—C1—C2	124.2 (2)	C24—C25—C26	119.5 (2)
O1—C1—C6	115.8 (2)	C24—C25—H25	120.227
C2—C1—C6	120.0 (2)	C26—C25—H25	120.2259
C1—C2—C3	120.3 (2)	O8—C26—C21	115.7 (2)
C1—C2—H2	119.8743	O8—C26—C25	124.6 (2)
C3—C2—H2	119.8742	C21—C26—C25	119.7 (2)
C2—C3—C4	120.3 (3)	O8—C27—C28	109.1 (2)
C2—C3—H3	119.8663	O8—C27—H27a	109.4717
C4—C3—H3	119.866	O8—C27—H27b	109.4708
C3—C4—C5	120.0 (2)	C28—C27—H27a	109.4709
C3—C4—H4	120.0034	C28—C27—H27b	109.4711
C5—C4—H4	120.0038	H27a—C27—H27b	109.8839
C4—C5—C6	119.9 (2)	O9—C28—C27	109.5 (2)
C4—C5—H5	120.0572	O9—C28—H28a	109.4715
C6—C5—H5	120.0574	O9—C28—H28b	109.4712
O2—C6—C1	115.6 (2)	C27—C28—H28a	109.4707
O2—C6—C5	124.9 (2)	C27—C28—H28b	109.4711
C1—C6—C5	119.6 (2)	H28a—C28—H28b	109.4873
O2—C7—C8	108.6 (2)	O9—C29—C30	109.9 (2)
O2—C7—H7a	109.4716	O9—C29—H29a	109.4711
O2—C7—H7b	109.471	O9—C29—H29b	109.4712
C8—C7—H7a	109.4713	C30—C29—H29a	109.4714
C8—C7—H7b	109.4716	C30—C29—H29b	109.4712
H7a—C7—H7b	110.3703	H29a—C29—H29b	109.0288
O3—C8—C7	109.0 (2)	O10—C30—C29	108.7 (2)
O3—C8—H8a	109.4704	O10—C30—H30a	109.4715
O3—C8—H8b	109.4715	O10—C30—H30b	109.4713
C7—C8—H8a	109.4715	C29—C30—H30a	109.4711
C7—C8—H8b	109.4715	C29—C30—H30b	109.4714
H8a—C8—H8b	109.952	H30a—C30—H30b	110.27
O3—C9—C10	109.7 (2)	O10—C31—C32	124.3 (2)
O3—C9—H9a	109.471	O10—C31—C36	116.3 (2)
O3—C9—H9b	109.4715	C32—C31—C36	119.4 (2)
C10—C9—H9a	109.4714	C31—C32—C33	120.1 (2)
C10—C9—H9b	109.4705	C31—C32—H32	119.973
H9a—C9—H9b	109.2636	C33—C32—H32	119.9722
O4—C10—C9	109.2 (2)	C32—C33—C34	120.2 (3)
O4—C10—H10a	109.4713	C32—C33—H33	119.8784
O4—C10—H10b	109.4713	C34—C33—H33	119.8778
C9—C10—H10a	109.4712	C33—C34—C35	120.3 (3)
C9—C10—H10b	109.4714	C33—C34—H34	119.8372
H10a—C10—H10b	109.7634	C35—C34—H34	119.8381
O4—C11—C12	124.5 (2)	C34—C35—C36	120.1 (3)
O4—C11—C16	116.1 (2)	C34—C35—H35	119.9488
C12—C11—C16	119.3 (2)	C36—C35—H35	119.9493
C11—C12—C13	120.0 (2)	O11—C36—C31	115.8 (2)
C11—C12—H12	120.0012	O11—C36—C35	124.4 (2)
C13—C12—H12	119.9988	C31—C36—C35	119.8 (2)
C12—C13—C14	120.5 (3)	O11—C37—C38	108.6 (2)
C12—C13—H13	119.7378	O11—C37—H37a	109.4712
C14—C13—H13	119.7376	O11—C37—H37b	109.4723
C13—C14—C15	119.8 (2)	C38—C37—H37a	109.4709
C13—C14—H14	120.0818	C38—C37—H37b	109.4708
C15—C14—H14	120.0821	H37a—C37—H37b	110.3319
C14—C15—C16	119.6 (2)	O12—C39—C40	109.2 (2)
C14—C15—H15	120.1961	O12—C39—H39a	109.4712
C16—C15—H15	120.1939	O12—C39—H39b	109.4706
O5—C16—C11	115.6 (2)	C40—C39—H39a	109.4708
O5—C16—C15	123.7 (2)	C40—C39—H39b	109.4716
C11—C16—C15	120.7 (2)	H39a—C39—H39b	109.7323
O5—C17—C18	108.1 (2)	O12—C38—C37	110.4 (2)
O5—C17—H17a	109.4708	O12—C38—H38a	109.471
O5—C17—H17b	109.471	O12—C38—H38b	109.4713
C18—C17—H17a	109.4713	C37—C38—H38a	109.4714
C18—C17—H17b	109.4714	C37—C38—H38b	109.4718
H17a—C17—H17b	110.8097	H38a—C38—H38b	108.4738
O6—C18—C17	110.1 (2)	O7—C40—C39	107.8 (2)
O6—C18—H18a	109.4711	O7—C40—H40a	109.4719
O6—C18—H18b	109.4712	O7—C40—H40b	109.4709
C17—C18—H18a	109.4709	C39—C40—H40a	109.4714
C17—C18—H18b	109.4714	C39—C40—H40b	109.4709
H18a—C18—H18b	108.827	H40a—C40—H40b	111.0734
			
?—?—?—?	?		

### Hydrogen-bond geometry (Å, °)

**Table:**

Cg1, Cg2, Cg3 and Cg4 are the centroids of the C31–C36, C11–C16, C21–C26 and C1–C6 rings, respectively.

**Table:**

*D*—H···*A*	*D*—H	H···*A*	*D*···*A*	*D*—H···*A*
O21—H1···O3	1.14 (3)	1.64 (3)	2.763 (3)	168 (3)
O21—H1···O4	1.14 (3)	2.39 (3)	2.835 (3)	101 (2)
O21—H7···O17	1.22 (4)	1.73 (4)	2.945 (4)	171 (3)
O22—H8···O9	1.20 (3)	1.66 (3)	2.802 (3)	157 (3)
O22—H8···O10	1.20 (3)	2.29 (3)	2.837 (3)	104.4 (19)
O21—H9···O4	1.09 (3)	2.40 (3)	2.835 (3)	102 (2)
O21—H9···O5	1.09 (3)	1.87 (3)	2.910 (3)	159 (3)
O21—H9···O6	1.09 (3)	2.47 (4)	2.967 (3)	107 (2)
O22—H10···O11	1.05 (3)	1.90 (3)	2.840 (3)	149 (3)
O22—H10···O12	1.05 (3)	2.34 (3)	2.895 (3)	112 (2)
C5—H5···O18^i^	0.96	2.52	3.479 (3)	177
C8—H8b···O19	0.96	2.55	3.416 (3)	150
C15—H15···O13^ii^	0.96	2.42	3.369 (3)	169.02
C20—H20b···O16^iii^	0.96	2.43	3.165 (3)	134
C35—H35···O15^ii^	0.96	2.59	3.278 (4)	129
C38—H38b···O19^iv^	0.96	2.50	3.447 (3)	168
C17—H17a···Cg1	0.96	2.87	3.704 (3)	146
C37—H37b···Cg2^v^	0.96	2.99	3.825 (3)	146
C13—H13···Cg3	0.96	3.20	4.070 (3)	150
C33—H33···Cg4^v^	0.96	3.00	3.899 (3)	156

Symmetry codes: (i) *x*+1, *y*, *z*; (ii) *x*, −*y*+1/2, *z*−1/2; (iii) −*x*+1, *y*+1/2, −*z*+1/2; (iv) *x*−1, −*y*+1/2, *z*−1/2; (v) *x*−1, *y*, *z*.
